# Supplementary material for: Behavioral predictors associated with HIV screening needs in gay Korean men during the COVID-19 pandemic
Source: PLoS One. 2023 Jun 12;18(6):e0287061. doi: 10.1371/journal.pone.0287061 (PMC10259784; doi:10.1371/journal.pone.0287061)
Supplement: S1 File — (PDF) [file pone.0287061.s001.pdf]

코로나-19 판데믹 기간 성 소수자의 HIV 검진과 성 건강에 대한 조사 연구

A. 지식, 태도, 신념

A1. 귀하는 이반씨티 싸이트를 얼마나 이용하십니까?
① 매일　② 이틀에 한번　③ 3~4일에 한번　④ 일주일에 한번　⑤ 한 달에 한두 번

A2. 귀하는 **하루 평균** 아래의 미디어를 각각 얼마나 사용하십니까?

		전혀 사용하지 않음	10분 미만	10분 이상-30분 미만	30분 이상-60분 미만	1시간 이상 - 2시간 미만	2시간 이상 - 3시간 미만	3시간 이상 - 5시간 미만	5시간 이상
1	텔레비전 시청	①	②	③	④	⑤	⑥	⑦	⑧
2	라디오 청취	①	②	③	④	⑤	⑥	⑦	⑧
3	종이 신문 읽기	①	②	③	④	⑤	⑥	⑦	⑧
4	스마트폰으로 인터넷 검색	①	②	③	④	⑤	⑥	⑦	⑧
5	컴퓨터로 인터넷 검색	①	②	③	④	⑤	⑥	⑦	⑧

A3. 귀하는 평소 얼마나 적극적으로 **건강정보**를 찾는 편이십니까?
① 매우 열심히 찾는다　② 열심히 찾는 편이다　③ 보통이다
④ 찾지 않는 편이다　⑤ 거의 찾지 않는다

A4. 귀하는 게이바를 얼마나 자주 이용하십니까?
① 전혀 안 간다　② 한 달에 한번 정도　③ 한 달에 2-3번　④ 일주일에 한번 이상

A5. 귀하는 커밍 아웃을 하였습니까?
① 자발적으로 하였다　② 타의로 하였다　③ 하지 않았다

A6. 귀하는 성 소수자로서 자신의 의견을 공개적으로 표현하거나 의사소통을 하는데 얼마나 자유롭거나 억압을 받는다고 느끼십니까? 1점(매우 억압됨)부터 10점(매우 자유로움) 사이에서 골라 표시해 주십시오.

매우 억압되어 있다					보통				매우 자유롭다
[1]	[2]	[3]	[4]	[5]	[6]	[7]	[8]	[9]	[10]

A7. 귀하는 동성애자에 대한 사회적 차별문제에 대해 어떻게 생각하십니까?

	매우 그렇다	그렇다	그저 그렇다	그렇지 않다	전혀 그렇지 않다
1) 동성애자는 취업이나 승진에 있어서 차별을 받는다고 생각한다	[1]	[2]	[3]	[4]	[5]
2) 동성애자는 일반 사람들보다 덜 존중 받는다고 생각한다	[1]	[2]	[3]	[4]	[5]
3) 일반 사람들이 동성애자를 두려워한다고 생각한다	[1]	[2]	[3]	[4]	[5]
4) 동성애자는 일반인에게 놀림이나 모욕을 당한다고 생각한다	[1]	[2]	[3]	[4]	[5]

A8. 귀하는 다음의 질문에 대해서 동의하십니까? 혹은 반대하십니까? 감염인을 본 적이 없더라도 주변에 있다고 가정해서 답해 주십시오.

	매우 그렇다	그렇다	그저 그렇다	그렇지 않다	전혀 그렇지 않다
1) 내 가족 중에 HIV 감염인이 생긴다면 집에서 함께 지낼 수 있다	[1]	[2]	[3]	[4]	[5]
2) HIV 감염인이 같은 직장에 다닌다면 사표를 내도록 해야 한다	[1]	[2]	[3]	[4]	[5]
3) HIV에 감염된다면 그것은 그 사람의 책임이다	[1]	[2]	[3]	[4]	[5]
4) HIV 감염인은 다른 사람과 격리시켜 수용시설에 보내야 한다	[1]	[2]	[3]	[4]	[5]
5) 나는 HIV 감염인과 함께 식사를 할 수 있다	[1]	[2]	[3]	[4]	[5]

A9. 귀하는 대부분의 일반 사람들을 믿을 수 있다고 생각하십니까? 아니면 조심해야 한다고 생각하십니까?
① 믿을 수 있다 ② 조심해야 한다 ③ 모르겠다
A10. 귀하는 대부분의 동성애자들을 믿을 수 있다고 생각하십니까? 아니면 조심해야 한다고 생각하십니까?
① 믿을 수 있다 ② 조심해야 한다 ③ 모르겠다

A11. 귀하는 다른 동성애자들과 비교할 때 자신이 HIV에 감염될 가능성은 얼마나 된다고 보십니까?
① 매우 높다 ② 높은 편이다 ③ 보통이다 ④ 낮은 편이다 ⑤ 매우 낮다

A12. 귀하는 본인의 성적 정체성을 어떻게 규정하십니까?
① 이성애자 ② 동성애자 ③ 양성애자 ④ 기타 ⑤ 생각해 본 적 없음(혹은 모름)

B. HIV 검진

B1. 귀하는 지금까지 한번이라도 HIV 검사를 받은 경험이 있습니까? 귀하의 뜻과 상관없이 종합건강검진, 수술이나 치료, 헌혈 시 HIV 검사가 포함된 경우도 해당됩니다.
① 예(===>B1-1로 이동) ② 아니오(===>B2로 이동)

B1-1. <u>가장 최근</u>에 HIV 검사를 받은 것은 언제입니까?
① 올해(2022년) 받았음
② 작년(2021년)에 받았음
③ 재작년(2020년)에 받았음
④ 2020년 코로나-19 발생 이후로는 HIV 검사를 받지 않았음

B1-2. 그때 HIV 검사를 받은 장소는 어디였습니까?
① 병원 ② 보건소 ③ 동성애자 검진상담소(iSHAP) ④ 외국에 있는 검사기관
⑤ 적십자/한마음 혈액원 ⑥ 기타

B1-3. 귀하는 이름이나 주민번호 등의 개인정보를 밝히지 않고 익명으로 보건소나 AIDS 검진 상담소 등에서 HIV 검진을 받을 수 있다는 사실을 알고 계십니까?
① 알고 있다 ② 모른다

B2. 귀하는 2020년 코로나-19 발생 이후 HIV 검사를 <u>받고 싶었으나 받지 못한 적</u>이 있습니까?
① 있다 ② 없다

B3. 귀하는 <u>올해</u> HIV 검사를 받아보실 의향이 있으십니까?
① 있다(===>C1으로 이동) ② 없다(===>B3-1로 이동)

B3-1. 귀하께서 올해 HIV 검사를 받을 생각이 없다면 주된 이유는 무엇입니까?
① HIV 감염을 걱정할 행동을 하지 않았으므로
② 검사하러 갔다가 코로나-19에 감염될까봐서
③ 검사결과가 어떻게 나올지 두려워서
④ 검사소까지 너무 멀어서
⑤ 비용이 들 것 같아서
⑥ 신분 노출이 우려되어서
⑦ 시간이 없어서
⑧ 기타

C. 성 건강

C1. 평소 성관계를 할 때 콘돔을 얼마나 사용하십니까?
① 매번(100% 사용)
② 자주 사용(50%~90% 정도 사용)
③ 가끔 사용(10%~50% 정도 사용)
④ 거의 사용하지 않음(10% 미만 사용)
⑤ 모르겠다

C2. 귀하는 애인관계처럼 고정적으로 성관계를 하는 동성의 파트너가 있습니까?
① 있다　　② 없다

C3. 귀하는 지난 1년 동안 성관계로 감염되는 질병을 진단 받거나 치료한 적이 있습니까?
① 있다(===>C3-1로 이동)　　② 없다(===>C4로 이동)

C3-1. 어떤 질병이었습니까? 해당사항에 모두 체크해 주십시오.
① 임질
② 매독
③ 클라미디아
④ 성기 단순포진
⑤ HIV/AIDS
⑥ 기타 성병

C4. 귀하는 항문성교를 선호하십니까?
① 선호한다　　② 선호하지 않는다

D. 건강행태와 건강수준

D1. 귀하는 현재 담배를 피우십니까?
① 그렇다(흡연자)　　② 아니다(비흡연자)

D2. 귀하는 평소에 술을 얼마나 자주 마십니까?
① 전혀 마시지 않는다(===>D3으로 이동)
② 한 달에 1번 이하(===>D3으로 이동)
③ 한 달에 2~4번 정도(===>D2-1로 이동)
④ 일주일에 2~3번 정도(===>D2-1로 이동)
⑤ 일주일에 4번 이상(===>D2-1로 이동)

D2-1. 술을 마시는 날은 보통 몇 잔을 마십니까?
① 소주 1~2잔 또는 맥주 1병
② 소주 3~4잔 또는 맥주 2병
③ 소주 5~6잔 또는 맥주 3병
④ 소주 7잔 이상 또는 맥주 4병 이상

D3. 생활의 만족이나 삶의 질을 전반적으로 고려할 때 귀하는 현재 얼마나 행복하십니까? 또는 불행하십니까?
① 매우 행복하다 ② 행복한 편이다 ③ 행복하지도 불행하지도 않다 ④ 불행한 편이다 ⑤ 매우 불행하다

D4. 귀하의 현재 건강상태는 어떻습니까?
① 매우 좋다 ② 좋은 편이다 ③ 보통이다 ④ 나쁜 편이다 ⑤ 매우 나쁘다

D5. 귀하는 주변에 HIV에 감염된 사람이 있습니까?
① 있다 ② 없다

D6. 귀하는 HIV에 감염되었습니까?
① 그렇다 ② 아니다

D7. 귀하는 성병이나 HIV/AIDS 이외에 기저질환이 있습니까?
① 있다(===>D7-1로 이동) ② 없다(===>E1로 이동)

D7-1. 귀하께서 가지고 있는 성병이나 HIV/AIDS 이외의 기저질환은 무엇입니까? (복수응답)
① 결핵
② 당뇨병
③ 심장질환
④ 뇌혈관질환
⑤ 암(과거에 치료받은 경우도 포함)
⑥ 만성간염 또는 간경변
⑦ 천식 등 호흡기 질환
⑧ 자가면역질환(갑상선, 류마티스, 대장성궤양 등)
⑨ 정신질환(우울증 등)
⑩ 기타

E. 코로나-19와 건강보호

E1. 귀하는 코로나-19 백신을 몇 차까지 접종하셨습니까?
① 한번도 접종하지 않았다(===>E4로 이동)
② 1차(===>E2로 이동)
③ 2차(===>E2로 이동)
④ 3차 이상(===>E2로 이동)

E2. 귀하가 맞은 백신의 종류는 무엇이었습니까? 모두 체크해주세요. (복수응답)
① 아스트라제네카
② 화이자
③ 모더나
④ 얀센
⑤ 코백스
⑥ 잘 모르겠음

E3. 코로나 백신 투여 후 이상 증상 혹은 부작용을 경험했습니까?
① 특별한 증상 혹은 부작용은 없었다
② 경미한 몸살 증상은 있었지만 바로 없어졌다
③ 일상 생활에 지장을 줄 정도의 비교적 심한 증상 및 부작용이 있었다
④ 입원을 할 정도로 꽤 심한 증상 및 부작용이 있었다
⑤ 기타(구체적으로 입력)

E4. 귀하는 코로나-19 시국에 다음과 같은 질병 예방 수칙을 얼마나 실천하셨습니까?

		전혀 실천하지 않는다	거의 실천하지 않는다	보통이다	가급적 실천한다	반드시 실천한다
1	손 씻기 혹은 손 세정제 사용하기	①	②	③	④	⑤
2	전염병에 감염된 사람과의 접촉을 피하기	①	②	③	④	⑤
3	눈, 코, 입 등을 손으로 만지지 않기	①	②	③	④	⑤
4	전염병 유행 시 외출 자제하기	①	②	③	④	⑤
5	사람들과의 접촉이 빈번한 장소 피하기	①	②	③	④	⑤
6	마스크 착용하기	①	②	③	④	⑤

E5. 귀하는 코로나-19에 감염된 적이 있습니까? (현재 감염상태인 경우도 포함)
① 있다(===>E5-1로 이동) ② 없다(===>M1로 이동)

E5-1. 귀하께서 코로나-19에 감염된 시기는 언제입니까?
① 2020년 ② 2021년 ③ 2022년

M. 응답자의 일반적 특성

M1. 귀하의 최종학력은 어떻게 되십니까?
① 중학교 졸업 이하
② 고등학교 졸업
③ 대학교 졸업
④ 대학원 재학 이상

M2. 귀하는 현재 여성과 혼인관계에 있습니까?
① 예 ② 아니오

M3. 귀하는 현재 소득이 발생하는 경제활동에 종사하고 계십니까?
① 예(===>M3-1로 이동)
② 아니오(===>M4로 이동)

M3-1. 귀하의 종사상 지위는 무엇입니까?
① 상시근로자
② 임시근로자
③ 자영업자

M4. 임금, 월급, 사회보장 또는 은퇴 연금, 친척의 도움 등 모든 소득을 포함하여 **귀하**의 작년(2021년) **총합계 수입(연봉)**은 얼마입니까? 세전 수입으로 말씀해 주십시오.
① 1,000만원 미만 ② 1,000만원 이상 ~ 2,000만원 미만

③ 2,000만원 이상 ~ 3,000만원 미만
④ 3,000만원 이상 ~ 4,000만원 미만
⑤ 4,000만원 이상 ~ 5,000만원 미만
⑥ 5,000만원 이상 ~ 6,000만원 미만
⑦ 6,000만원 이상 ~ 7,000만원 미만
⑧ 7,000만원 이상 ~ 8,000만원 미만
⑨ 8,000만원 이상 ~ 9,000만원 미만
⑩ 9,000만원 이상 ~ 1억 미만
⑪ 1억 이상

- 설문에 응해주셔서 대단히 감사합니다! -
