# Supplementary material for: Behavioral predictors associated with HIV screening needs in gay Korean men during the COVID-19 pandemic
Source: PLoS One. 2023 Jun 12;18(6):e0287061. doi: 10.1371/journal.pone.0287061 (PMC10259784; doi:10.1371/journal.pone.0287061)
Supplement: Attachment — Submitted filename: PONE-D-22-34866_reviewer.docx [file pone.0287061.s002.docx]

PONE-D-22-34866

Behavioral Predictors Associated with HIV Screening Needs in Gay Korean Men during the COVID-19 Pandemic

| **No** | **Reviewers' Comments** | **Author response** |
| --- | --- | --- |
|  | Reviewer:  This is a good study reporting behavioral charateristics among MSM during pandemic. This is very importance baseline data to update policy on accessibility or availability of appropriate HIV screening services to MSM during pandemic. |  |
|  | Page 8 line: [The time taken to complete the self-administered questionnaire was approximately 20 minutes.]  I would suggest to authors to explain details on self-administered questionnaire in the Methods. Is it validated self-administered questionnaire? Which previous study using this validated self-administered questionnaire? If not using validated self-administered questionnaire, any validation done prior to current study? Example of validated self-administered questionnaire need to upload in the full paper as appendix or supporting document |  |
