# Supplementary material for: Behavioral predictors associated with HIV screening needs in gay Korean men during the COVID-19 pandemic
Source: PLoS One. 2023 Jun 12;18(6):e0287061. doi: 10.1371/journal.pone.0287061 (PMC10259784; doi:10.1371/journal.pone.0287061)
Supplement: Attachment — Submitted filename: Response to reviewer comments.docx [file pone.0287061.s003.docx]

**Point-by-point responses to reviewers’ comments**

Ref.: PONE-D-22-34866

Behavioral Predictors Associated with HIV Screening Needs in Gay Korean Men during the COVID-19 Pandemic

Dear Dr. Omid Dadras, Academic Editor of PLOS ONE

Thank you for your letter dated May 10, 2023. We would like to express our gratitude to the editor for making the decision to be a minor revision. In this revised manuscript, we have supplemented in detail the points pointed out by reviewers and editors. We also uploaded the datasets and questionnaires used in the study as supporting files. As a result, we believe that our manuscript now meets PLOS ONE's publication criteria. Provided below are the details of how we addressed the issues raised by the reviewers.

Very best,

Authors

**Reviewers’ Comments**

1. This is a good study reporting behavioral characteristics among MSM during pandemic. This is very importance baseline data to update policy on accessibility or availability of appropriate HIV screening services to MSM during pandemic.

>>> Authors’ Response: Thank you for recognizing the needs and strengths of our research. In this revision, the authors supplemented the manuscript to reveal the novelty of the study more clearly.

2. Page 8 line: [The time taken to complete the self-administered questionnaire was approximately 20 minutes.] I would suggest to authors to explain details on self-administered questionnaire in the Methods. Is it validated self-administered questionnaire? Which previous study using this validated self-administered questionnaire? If not using validated self-administered questionnaire, any validation done prior to current study? Example of validated self-administered questionnaire need to upload in the full paper as appendix or supporting document.

>>> Authors’ Response: The survey instruments and self-administered questionnaire of this study were verified for validity and reliability in "Developing the behavioral monitoring survey system for the high HIV risk group", a project supported by the Korea Federation for HIV/Prevention in 2011. At that time, the project aimed to develop behavioral monitoring indicators suitable for LGBTI people in Korea based on UNAIDS' "Core Indicators for National AIDS Programs" in 2007. The author of this paper participated in the project at the time and has been updating the survey tools and questionnaire while carrying out the project on homosexuals in Korea. However, in the past 10 years, project-related data were classified as confidential and could not be published as a academic thesis. Since the relevant information has now been declassified, at the request of the reviewer, we have uploaded the entire questionnaire as a supporting document.

3. Reviewer #1: Good work done. However, I wish you could cite more to support your findings in the discussion sections. Also, structure the strengths and limitations and conclusion sections with headings to make the work standout.

>>> Authors’ Response: Following the reviewer's recommendations, we additionally cited recent literature to support our article in the discussion section. Also, we divided the paragraphs to clearly reveal the strengths and limitations of the thesis.

4. Reviewer #2: The authors are to be congratulated for considering this important subject. The concept of the paper is good, starting from the introduction, methodology, discussion to a conclusion. The result of the paper supports the conclusion.

>>> Authors’ Response: Thank you for praising the value and originality of this study. In this revised manuscript, we highlighted the strengths and supplemented the weaknesses of this study. As a result, we believe that our manuscript is much stronger and clearer.

5. Reviewer #3: This is a good study reporting behavioral characteristics among MSM during pandemic. This is very importance baseline data to update policy on accessibility or availability of appropriate HIV screening services to MSM during pandemic. Applause to all authors.

>>> Authors’ Response: Thank you for praising the value and originality of this study. In this revised manuscript, we highlighted the strengths and supplemented the weaknesses of this study. As a result, we believe that our manuscript is much stronger and clearer.
